# Mechanistic insights into the role of the chemokine CCL2/CCR2 axis in dorsal root ganglia to peripheral inflammation and pain hypersensitivity

**DOI:** 10.1186/s12974-021-02125-y

**Published:** 2021-03-23

**Authors:** Marc-André Dansereau, Élora Midavaine, Valérie Bégin-Lavallée, Mounir Belkouch, Nicolas Beaudet, Jean-Michel Longpré, Stéphane Mélik-Parsadaniantz, Philippe Sarret

**Affiliations:** 1 Département de Pharmacologie & Physiologie, Institut de Pharmacologie de Sherbrooke, Faculté de médecine et des sciences de la santé, Université de Sherbrooke, 3001, 12e Avenue Nord, Sherbrooke, Quebec J1H 5N4 Canada; 2 Centre de Recherche Institut de la Vision, Université Pierre et Marie Curie, INSERM, UMR_S968, CNRS, UMR_7210, Paris, France

**Keywords:** Nociceptor, INCB3344, Substance P, Nociception, MCP-1, CFA, Formalin, Peripheral inflammation, Calcium mobilization, Dorsal root ganglion

## Abstract

**Background:**

Pain is reported as the leading cause of disability in the common forms of inflammatory arthritis conditions. Acting as a key player in nociceptive processing, neuroinflammation, and neuron-glia communication, the chemokine CCL2/CCR2 axis holds great promise for controlling chronic painful arthritis. Here, we investigated how the CCL2/CCR2 system in the dorsal root ganglion (DRG) contributes to the peripheral inflammatory pain sensitization.

**Methods:**

Repeated intrathecal (*i.t*.) administration of the CCR2 antagonist, INCB3344 was tested for its ability to reverse the nociceptive-related behaviors in the tonic formalin and complete Freund’s adjuvant (CFA) inflammatory models. We further determined by qPCR the expression of CCL2/CCR2, SP and CGRP in DRG neurons from CFA-treated rats. Using DRG explants, acutely dissociated primary sensory neurons and calcium mobilization assay, we also assessed the release of CCL2 and sensitization of nociceptors. Finally, we examined by immunohistochemistry following nerve ligation the axonal transport of CCL2, SP, and CGRP from the sciatic nerve of CFA-treated rats.

**Results:**

We first found that CFA-induced paw edema provoked an increase in CCL2/CCR2 and SP expression in ipsilateral DRGs, which was decreased after INCB3344 treatment. This upregulation in pronociceptive neuromodulators was accompanied by an enhanced nociceptive neuron excitability on days 3 and 10 post-CFA, as revealed by the CCR2-dependent increase in intracellular calcium mobilization following CCL2 stimulation. In DRG explants, we further demonstrated that the release of CCL2 was increased following peripheral inflammation. Finally, the excitation of nociceptors following peripheral inflammation stimulated the anterograde transport of SP at their peripheral nerve terminals. Importantly, blockade of CCR2 reduced sensory neuron excitability by limiting the calcium mobilization and subsequently decreased peripheral transport of SP towards the periphery. Finally, pharmacological inhibition of CCR2 reversed the pronociceptive action of CCL2 in rats receiving formalin injection and significantly reduced the neurogenic inflammation as well as the stimuli-evoked and movement-evoked nociceptive behaviors in CFA-treated rats.

**Conclusions:**

Our results provide significant mechanistic insights into the role of CCL2/CCR2 within the DRG in the development of peripheral inflammation, nociceptor sensitization, and pain hypersensitivity. We further unveil the therapeutic potential of targeting CCR2 for the treatment of painful inflammatory disorders.

## Introduction

The interaction between the immune and central nervous systems, through neuron-glia communication or immune cell infiltration, represents an expanding topic rallying the scientific community. Amongst the various immuno-inflammatory mediators, chemokines appear to behave as important modulators of brain functions. Initially identified and named for their chemotactic properties on immune cells (for review, see [1, 2]), accumulating studies pinpointed many roles for chemokines during brain development as well as in normal brain functions and various pathological conditions [3–6], making them key actors at both immune and nervous system levels [7].

Over the last decade, it has become more evident that immune cells, glia, and neurons form a complex regulatory network that is able to alter pain sensitivity and to mediate the transition from acute to chronic pain [8]. The monocyte chemoattractant protein 1 (formerly MCP-1, now named CCL2) and its main receptor CCR2 are amongst the most characterized neuroactive chemokine couple in respect to their ability to modulate nociception [9–12]. Growing evidence supports that CCR2 activation by CCL2 contributes to both acute and chronic pain behaviors. For instance, the spinal administration of CCL2 induces sustained painful mechanical hypersensitivity [13–15]. In addition, there is an abundant literature supporting the role of the CCL2/CCR2 axis in the regulation of nociceptive transmission, especially for the management of chronic neuropathic pain [11, 12, 16–18]. Accordingly, CCR2 is increased in neuropathic and HIV-associated chronic pain, and CCR2-KO mice do not develop mechanical allodynia following chronic constriction of the sciatic nerve [19–22]. In contrast, the role of the CCR2/CCL2 axis in regulating chronic inflammatory pain and peripheral inflammation remains poorly defined.

Pain is reported as the leading cause of disability in the two most common forms of arthritis, rheumatoid arthritis (RA), and osteoarthritis (OA), with 90% of patients consulting rheumatologists classifying pain as one of their three main priorities [23–25]. Although RA and OA are two very different conditions, they share some common symptoms, such as joint pain, stiffness, and swelling. Furthermore, while inflammation is usually associated with RA, there is now growing evidence indicating that OA is also tied to inflammation [26, 27]. Consequently, ongoing joint inflammation is thought to play a key role in peripheral and central pain sensitization in inflammatory arthritis [28]. Conventional pharmacological treatments used to manage chronic painful arthritis include paracetamol, oral or topical non-steroidal anti-inflammatory drugs (NSAIDs), opioids, and intraarticular corticosteroids [29, 30]. However, benefits from these medications are limited and not suitable for many OA and RA patients given their adverse effect profiles (e.g., gastrointestinal complications, cardiovascular risks or abuse, and dependence potential). Accordingly, a recent meta-analysis reporting 40 years of cases of knee OA concludes that opioids and oral NSAIDs fail to show pain-relieving effects over placebo [31, 32]. Disease-modifying antirheumatic drugs (DMARDs), such as methotrexate or anti-TNF monoclonal antibody biologics are also used to treat inflammatory forms of arthritis [33]. Although DMARD therapy indirectly manages pain symptoms by preserving joint tissues, a large proportion of patients still reports feeling pain [34]. Consequently, new pharmacological treatment options are needed to manage pain in patients dealing with inflammatory arthritis.

Chemokine and chemokine receptors, such as CCL2/CCR2 play a key role in the migration of monocytes/macrophages and T cells and are therefore potential targets in the treatment of chronic inflammatory disorders. In that respect, it was shown that CCL2 levels are higher in the joint synovial fluid, surrounding synovial tissue and peripheral blood of RA [35, 36] and OA [37, 38] patients. This is further accompanied by an increase in CCR2 expressing cells in synovial tissues [39–42]. Interestingly, CCL2 upregulation in synovial fluid and injured tissue is positively correlated with pain intensity [37, 43]. These clinical findings thus suggest that the persistent pain experienced by RA and OA patients is mainly driven by a peripheral input. Likewise, in preclinical models of arthritis, both CCL2 and CCR2 were found to be upregulated in joints and peripheral tissues and to drive monocyte/macrophage recruitment and inflammation [42, 44–46]. Furthermore, in rodents developing chronic inflammatory arthritis, pharmacological treatment with either a cleaved form of CCL2, a neutralizing CCL2 monoclonal antibody, or systemic small molecule CCR2 antagonists all exhibited reduced severity of the pathology by reducing local inflammation [47–49].

Given the important role played by the CCL2/CCR2 axis in peripheral inflammation, nociceptor sensitization, and nociceptive processing, we have investigated here the mechanisms by which the CCL2/CCR2 axis could induce inflammatory hypernociception. To achieve this goal, we first evaluated the analgesic potential of repeated intrathecal (*i.t*.) administration of the CCR2 antagonist, INCB3344 [49–51] in two painful peripheral inflammatory conditions, namely the tonic formalin test and the complete Freund’s adjuvant (CFA) model of chronic inflammatory pain. We further determined how the blockade of CCR2 affected the expression of CCL2/CCR2 couple and of the proalgesic neuropeptides, substance P (SP), and calcitonin gene-related peptide (CGRP) in primary sensory neurons from CFA-treated rats. Using dorsal root ganglion (DRG) explants, acutely dissociated sensory neurons and calcium mobilization assay, we also investigated if the peripheral inflammation and pharmacological inhibition of CCR2 modulated the release of CCL2 and sensitization of nociceptors. Finally, we studied the anterograde axonal transport of CCL2, SP, and CGRP from the sciatic nerve of CFA-treated animals to determine their relative contribution to peripheral inflammation.

## Methods

### Animals

Male Sprague-Dawley rats (200–225g, Charles River, St. Constant, Quebec, Canada) were maintained on a 12-h light/dark cycle, with access to food and water ad libitum. They were allowed at least 5 days to habituate to the housing facility prior to manipulation, and 1 h to the experimentation room before any experiment. All animal procedures were approved by the ethical committee for animal care at the Université de Sherbrooke, in compliance with the policies and directives of the Canadian Council on Animal Care and guidelines from the International Association for the study of Pain (IASP).

### Drugs and intrathecal injection

One hour prior to behavioral experiments, lightly anesthetized animals (isoflurane 5% Abbott Laboratories, Montreal, Quebec, Canada) received a 25-μl intrathecal (*i.t*.) injection between the L5 and L6 vertebrae of either 6% DMSO in 0.9% saline (vehicle), 3 μg/kg of CCL2 (Peprotech, Rocky Hill, NJ, USA) either alone or in combination with 45 μg/kg INCB3344 (Pfizer, Pure Substance Program), or 45 μg/kg INCB3344 alone. The procedure is performed under 1 min, once the animal is asleep.

### Tonic inflammatory pain

Rats were allowed to habituate 1 h to the apparatus for three consecutive days prior to testing. On the experimental day, animals received an intradermal injection of 50 μl of 1% formaldehyde (i.e., 2.5% formalin, Fisher Scientific, Montreal, QC, Canada) in the left hind paw 1 h after their *i.t.* injection. They were then placed in a clear plexiglass enclosure (30 × 30 × 30 cm) for a 1-h observation trial. Pain was assessed using the weighted score method [52, 53]. Briefly, a nociceptive mean score was determined for every 3-min period of the recording time by measuring the amount of time spent in each of four behavioral states: 0, the injected paw is comparable to the contralateral paw; 1, the injected paw has little or no weight placed on it; 2, the injected paw is elevated and is not in contact with any surface; and 3, the injected paw is licked, bitten, or shaken. Rats exhibited typical biphasic nociceptive behaviors during the 60-min observational period after formalin administration. The two distinct phases of spontaneous pain behaviors that occur in rodents are proposed to reflect a direct effect of formalin on sensory receptors (phase I occurring within the first 5–10 min) and a longer-lasting pain due to inflammation and central sensitization (phase II beginning about 20 min and continuing for at least 40 min). These 2 phases are separated by a period of quiescence, the interphase, which is characterized by active inhibition of the formalin-induced nociceptive behaviors. The total area under the curve (A.U.C.) for the inflammatory phase was calculated between 21 and 42 min.

### Chronic inflammatory pain

Complete Freund’s adjuvant (CFA) (Calbiochem, La Jolla, CA, USA) was complemented with 7 mg/mL of *mycobacterium butyricum* (Difco laboratories, Detroit, MI, USA), then emulsified 1:1 with saline 0.9%. Under light anesthesia (isoflurane 5%), rats received an intraplantar injection of 100 μl of the freshly emulsified mixture. Pain-related behaviors were assessed on the same animals before and on days 3, 8, 9, and 10 following CFA injection. Animals received an *i.t.* injection of either vehicle or 45 μg/kg INCB3344 on days 8, 9, and 10 1 h before behavioral examination. Sham animals received an intraplantar injection of 100 μl saline.

### Mechanical sensitivity

To measure mechanical sensitivity, a dynamic plantar esthesiometer (Stoelting (Ugo Basile), Illinois, USA) was used. The metal probe, placed underneath a mesh floor, was aimed at the plantar surface of the hind paw and triggered when the animals were standing firmly on the mesh. The probe exerted a linearly increasing pressure (3.33 g/s) and was automatically stopped when either it reached the threshold of 50 g (cut-off) or the animal withdrew its hind paw. Four measures were taken on each hind paw, alternatively. Animals were habituated to the apparatus for 3 days before testing.

### Weight bearing

Discomfort was measured by a dynamic weight bearing apparatus (Bioseb, Boulogne, France), as described previously [54, 55]. Briefly, animals were allowed to move freely for 5 min on a floor-instrumented enclosure (22 × 22× 30 cm) allowing independent measures of the weight bore by each limb in synchronicity with a video capture. Zones stable for at least 0.4 s and triggering at least three captors with over 1 g of stimulation, with one of them recording over 4 g, were considered for analysis. Animals were not acclimatized to the setup before the initial testing period to maximize exploration behaviors.

### Edema

To follow the evolution of the inflammatory response induced by CFA intraplantar administration, the volume of the rats’ hind paws was determined with a plethysmometer, using the inflexion point of the ankle joint as anatomical reference (Stoelting (Panlab), Illinois, USA). Based on Archimedes’ principle, the volume displaced by the limb provokes a change in the conductance of an adjacent platinum electrode further converted in ml.

### Isolation and primary cell culture of dorsal root ganglion (DRG) neurons

Cultures of dissociated sensory neurons from adult rat DRG were prepared as described previously [56]. Under terminal anesthesia, rats were decapitated, and L4–L6 lumbar DRGs were dissected under aseptic conditions, then incubated with 1 mg/ml of collagenase (Roche Diagnostics, Indianapolis, IN, USA) in 0.6% of glucose in PBS for 1 h 30 min followed by trypsin-EDTA treatment (0.025% W/V; Gibco, Montréal, Québec, Canada) for 30 min at 37 °C. To stop enzymatic digestion, a 10× volume of DMEM high-glucose was added. After triturating the ganglia using heat-polished Pasteur pipettes, the sensory neurons were centrifuged (3 min at 800 rpm) and resuspended in DMEM high glucose with equal volume of HAM’s medium mixture F12 (Wisent inc, St-Bruno, Québec, Canada) supplemented with 10% fetal bovine serum heat inactivated (FBS, Wisent inc, St-Bruno, Québec, Canada), 2% of penicillin and streptavidin (Gibco, Montréal, Québec, Canada), and 50 ng/ml of nerve growth factor (NGF, Sigma-Aldrich, St-Louis, MO, USA). Finally, neuronal cells were plated onto poly-d-lysine-laminin-coated coverslips (Sigma-Aldrich, St-Louis, MO, USA) mounted on cell culture dishes (Mattek Corporation, Ashland, MA, USA). Cells were maintained during 15 h for calcium imaging at 37 ^o^C in a water-saturated atmosphere with 5% CO_2_.

### Intracellular calcium imaging

DRG neuronal cells grown on coverslips were loaded with 2 μM of Fura2-AM (Invitrogen, Eugene, OR, USA) in Tyrode’s solution (Sigma-Aldrich, St-Louis, MO, USA) [in nM 12 NaHCO_3_, 6 d-glucose, and 10 HEPES] for 20 min at room temperature in the dark. Cells were then washed twice with 0.1% bovine serum albumin (BSA, EM Science, Darmstadt, Germany) and incubated 20 min in Tyrode’s solution to de-esterify the dye. Calcium responses were determined by measuring fluorescence intensity changes using an epifluorescence microscope equipped with Metafluor software (Olympus Canada, Markham, ON, Canada). Isolated Fura2-AM loaded cells were selected and real-time calcium responses were monitored by alternating the excitation wavelengths between 340 and 380 nm every second. Intracellular calcium levels were expressed as relative total fluorescence [%∆F/F_0_ = [(F_s_ − F_0_)/F_0_] × 100 : Changes in fluorescence (∆F), baseline (F_0_), and stimulated (F_s_) fluorescence].

### qPCR

Ipsilateral L4 to L6 DRGs were freshly extracted on day 10 post-CFA, 3 h following the last scheduled *i.t.* injection of vehicle or INCB3344. Tissue samples were immediately snap-frozen, and mRNA extraction was performed using the RNeasy® mini kit (Qiagen, Mississauga, ON, Canada). mRNA quality and quantity were then analyzed with a NanoDrop® 1000 spectrophotometer (Thermo Fisher Scientific, Wilmington, DE, USA). Reverse transcription was achieved with the TaqMan® Reverse Transcription Kit (Applieb Biosystems, Carlsbad, CAL, USA) with 400 ng RNA. Real-time reactions were done using TaqMan® Gene Expression Master Mix (Applied Biosystems) on a Rotor-Gene 3000 (Corbett Life Science, Kirkland, Quebec). Results for every TaqMan primers (CCR2 (Rn01637698_s1), CCL2 (Rn00580555_m1), CGRP (Rn01511354_m1), Tachykinin 1 (Rn01500392_m1), GFAP (Rn01460868_m1), IL-1β (Rn00580432_m1), TNF-α (Rn00562055_m1), iNOS (Rn00561646_m1), IL6 (Rn00561420_m1), and COX2 (Rn00568225_m1) were normalized to the ribosomal protein S18 (Rn01428915_g1) and analyzed by the relative standard curve method.

### DRG superfusion

Ipsilateral L4 to L6 DRG were freshly extracted on day 10 post-CFA, 3 h following the last scheduled *i.t.* injection of vehicle or INCB3344. Ganglia were maintained in artificial cerebrospinal fluid (aCSF, in mM: NaCl, 136; NaHCO_3_, 16.2; KCl, 5.4; NaH_2_PO_4_, 1.2; CaCl_2_, 2.2; MgCl_2_, 1.2; Na_2_SO_4_, 0.5; glucose, 5.0, adjusted to pH 7.3 by bubbling with an O_2_/CO_2_ mixture, 95:5, v/v). The three DRG were inserted in a thermostated (37^o^C) Plexiglas chamber. Samples were continuously superfused at a flow rate of 125 μL/min and allowed to equilibrate for 20 min before superfusate collection. Following the collection of five 0.5 mL fractions (20 min), KCl concentration was increased to 30 mM (and NaCl decreased to 111.4 mM) for 8 min to depolarize the DRG. Eleven fractions were collected following KCl depolarization. CCL2 levels for each fraction were evaluated in 96-wells Nunc Maxi-Sorp plates (VWR, Mississauga, ON, Canada) by BD OptEIA™ Set Rat MCP-1 ELISA (BD Biosciences, Mississauga, ON, Canada) according to manufacturer’s instructions.

### Sciatic nerve ligation

A tight nerve ligation was performed on the ipsilateral sciatic nerve 8 days following CFA administration. Under isoflurane anesthesia (2% in O_2_; Abbott Laboratories, Montreal, QC, Canada), the ipsilateral common sciatic nerve was exposed at mid-thigh level and a single tight ligature (5.0 prolene suture) was performed proximal to the trifurcation. The skin was sealed with two 4.0 silk sutures. Animals were fixed 48 h later by intracardiac perfusion with 500 ml of paraformaldehyde 4% in 0.1 M phosphate buffer, pH 7.4. Animals received daily *i.t.* injection of 45 μg/kg INCB3344 starting 8 h before sciatic nerve ligation.

### Immunohistochemistry

Sciatic nerves were post-fixed at 4 °C for 24 h and cryoprotected in 0.1 M PBS containing 30% sucrose at 4 °C for another 24 h. The tissues were frozen at −20 °C in O.C.T. Compound (Sakura Finetek U.S.A., Inc., Torrance, USA), cryo-sectioned longitudinally at 20 μm and mounted on gelatin-coated pre-cleaned microscopic slides. Sections were washed in PBS (twice) and blocked in 0.2% triton X-100, 5% normal goat serum, and 2% albumin in PBS for 1 h at room temperature. Sections were then incubated in 0.1 M glycine solution and anti-CCL2 (polyclonal rabbit anti-MCP-1, 1:500, Torrey Pines Biolabs Inc., Secaucus, NJ, USA), or anti-substance P (polyclonal guinea-pig anti-substance P, 1:500 Neuromics, Edina, MN, USA) primary antibodies were incubated overnight at 4 °C. Sciatic nerve sections were washed twice and incubated in appropriate secondary fluorescent antibody, goat anti-rabbit IgG Alexa Fluor™-488, or goat anti-guinea-pig IgG Alexa Fluor™-488 (1:500, Invitrogen Molecular Probe, Burlington, ON, Canada) for 1 h at room temperature. Tissue sections were mounted with Aqua-Poly/Mount (Polysciences Inc., Warrington, USA). The specificity of each assay was determined by omitting the primary or secondary antibody.

Using a Leica DM4000B epifluorescence microscope (Leica Microsystems, Toronto, Canada), images were acquired using the same acquisition parameters (gain and exposure time). Captured images were analyzed using MetaMorph Offline Software. For each animal, 10 randomly selected sections were used for CCL2 or substance P fluorescence quantification. Regions of interest consisted in 600 μm next to the central side of the nerve ligation. Three rats per experimental condition were analyzed.

### Statistical analysis

One-way or two-way analysis of variance followed by Bonferroni post-test to account for appropriate comparisons were used to analyze behavioral measurement and CCL2 release. The percentage of inhibition of mRNA increase measured by qPCR was analyzed with a Wilcoxon signed-rank test since a Gaussian distribution could not be assumed by d’Agostino and Pearson omnibus normality test. Calcium mobilization induced by CCL2 in CFA animals compared to naïve rats was evaluated by one-way ANOVA followed by Bonferroni post-test. The inhibitory effect of INCB3344 on calcium mobilization was assessed by a Student’s *t* test. Statistical analyzes were performed by GraphPad Prism 7.0 (GraphPad software Inc, San Diego, CA, USA). A *P* value under 0.05 was considered significant.

## Results

### Formalin-induced tonic inflammatory pain

**Fig. 1:**
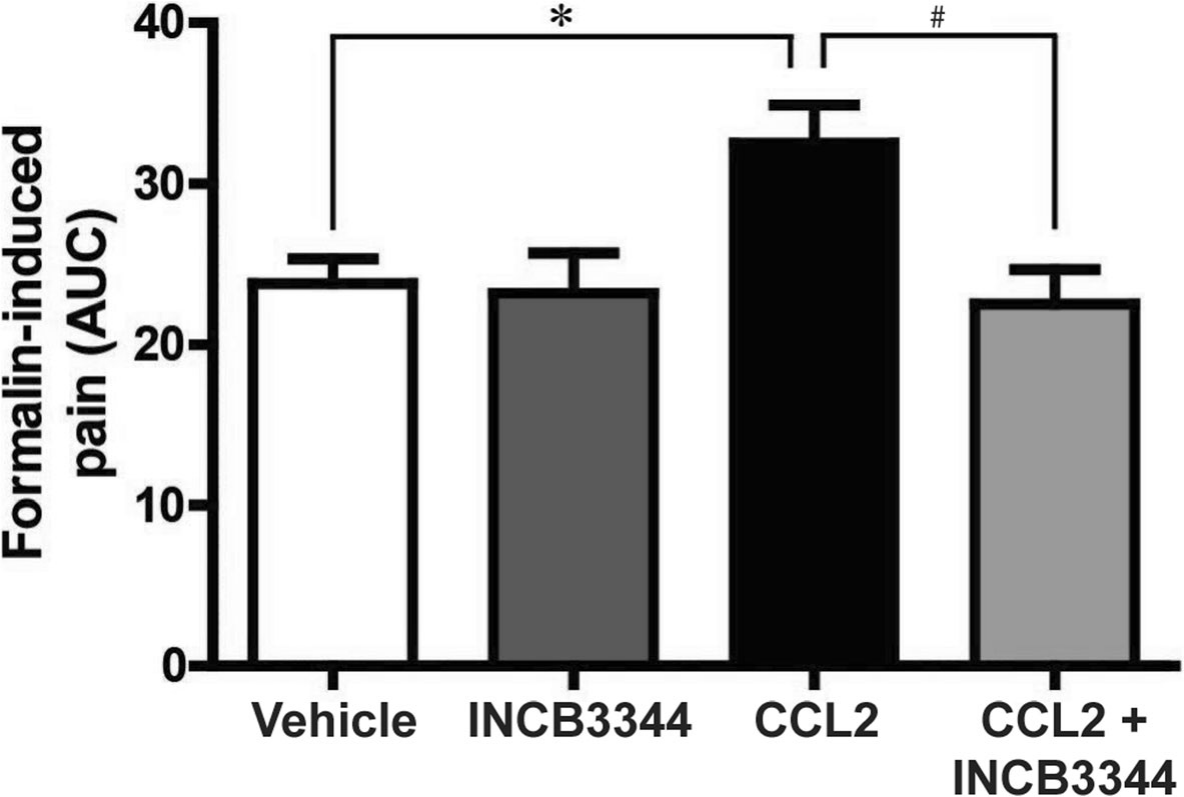
The chemokine CCL2 induces a CCR2-dependent pain hypersensitivity in the formalin model of persistent pain. Intrathecal administration of vehicle (DMSO 6%), 3 μg/kg CCL2 or 45 μg/kg INCB3344 with or without CCL2 is performed 1h prior to intraplantar formaldehyde (1%). The cumulative nociceptive response, expressed as mean area under the curve (A.U.C.), is measured during the inflammatory phase. Data correspond to mean ± SEM (*n* = 8). Asterisks denote statistical significance (*^, #^*P* < 0.05; ANOVA followed by Bonferroni’s multiple comparison test)

In order to determine the contribution of the CCL2-CCR2 chemokine axis to the development of pain associated with inflammatory conditions, we first investigated whether spinally administered CCL2 and/or the CCR2 antagonist, INCB3344 affected the nociceptive behaviors observed in the chemically induced formalin tonic pain model. Our results revealed that a 3 μg/kg i.t. injection of CCL2 exacerbates the nociceptive behaviors evoked by an intraplantar administration of 1% formaldehyde during the inflammatory phase of the formalin test (Fig. 1). Rats receiving a single i.t. injection of INCB3344 (45 μg/kg) alone did not exhibit reduction of their pain behaviors. However, co-administration of the CCR2 antagonist INCB3344 with CCL2 completely prevented CCL2-induced pain hypersensitivity to a mild formaldehyde injection (*P* < 0.05), indicating that CCL2 elicited pain facilitation via a CCR2-dependent mechanism.

### Intrathecal injection of a CCR2 antagonist reverses mechanical hypersensitivity and movement-evoked pain in rats receiving CFA

**Fig. 2:**
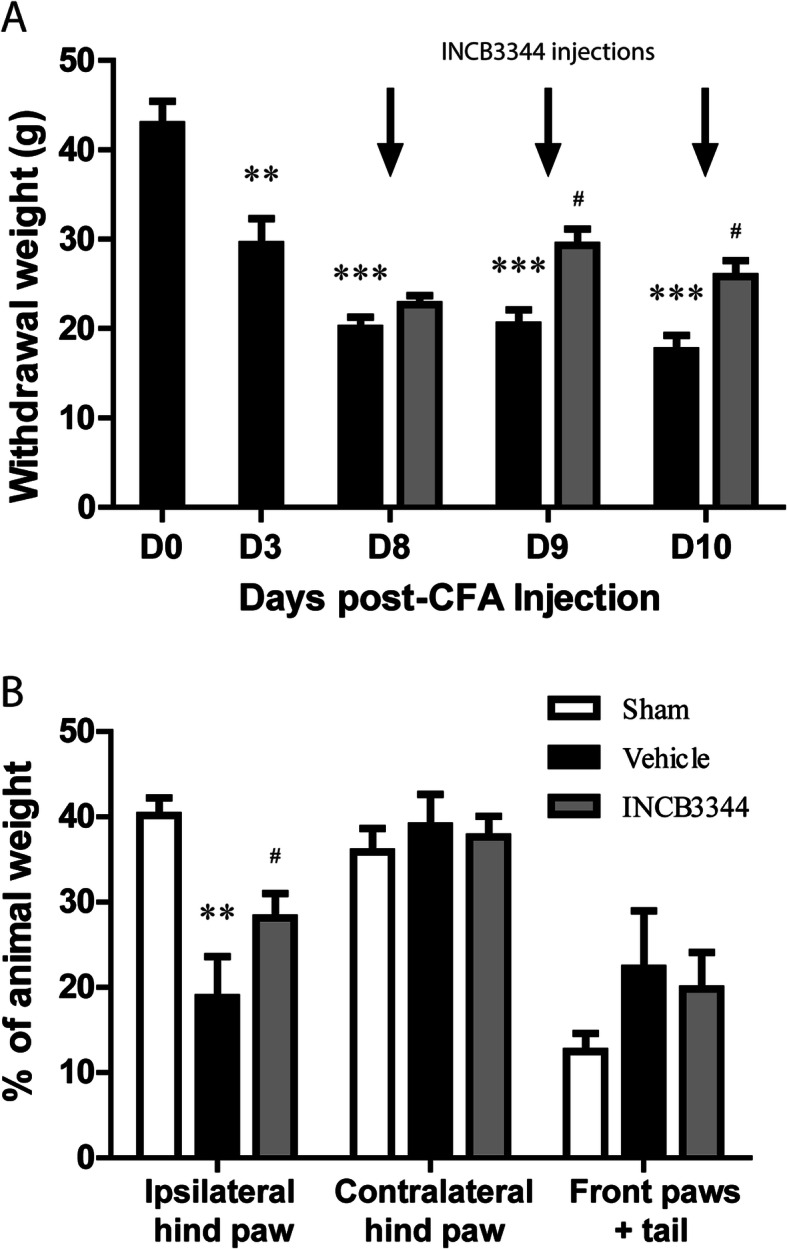
Effect of CCR2 antagonism on pain-related behaviors of CFA-treated rats. Animals received an intraplantar injection of 100 μL CFA with 4 mg/mL of *M. butyricum* in the left hind paw. Rats were treated daily by *i.t.* administration of 45 μg/kg INCB3344 starting at day 8 post-CFA (arrows). Pain was assessed 1-h post-treatment using (**a**) an automated von Frey test on days 3, 8, 9, and 10 post-CFA, and (**b**) a dynamic weight-bearing apparatus only at day 10 post-CFA. * relates to baseline or sham compared to vehicle (DMSO 6%), # to vehicle *versus* INCB3344. Data shown correspond to mean ± SEM (*n* = 8 for each group). *^, #^*P* < 0.05, **P < 0.01, ***P < 0.001; two-way ANOVA followed by Bonferroni post-tests

To further address the role of CCR2 in the development of chronic inflammatory pain, we next examined the effect of INCB3344 on the nociceptive behaviors evoked by unilateral injection of complete Freund’s adjuvant (CFA) into the plantar surface of the rat’s hind paw. Intraplantar injection of CFA provoked a massive unilateral inflammatory reaction that pertains over 10 days. This inflammation was accompanied by a progressive and sustained hypersensitivity to a non-painful mechanical stimulation elicited by a von Frey filament that began as early as day 3, stabilized at day 8 and lasted at least up to day 10 post-CFA injection (*P* < 0.01, Fig. 2a). No modification of the pain threshold was observed on the contralateral side (not shown). Intrathecal injection of 45 μg/kg INCB3344 exerted no pain relief on the first day of treatment (D8 post-CFA). However, repeated treatment increased the weight required to elicit a painful withdrawal by 44% at day 9 and 47% at day 10 (*P* < 0.05), thus producing anti-allodynic effects.

Concomitantly, CFA-injected animals exhibited a decrease in the weight bore on their injured limb, of approximately 50% after 10 days (Fig. 2b, *P* < 0.01). Interestingly, this shift in weight bearing was not accompanied by an increased percentage of the body weight resting on the contralateral hind paw, but rather on the forepaws. The time course of these movement-evoked pain-related behaviors closely resembles that of the hypersensitivity to mechanical stimuli. Importantly, this reduction of weight bearing observed on the ipsilateral hind limb was reversed following repeated INCB3344 treatment, with a maximal effect of 43% (*P* < 0.05, D10 post CFA, Fig 2b).

**Fig. 3:**
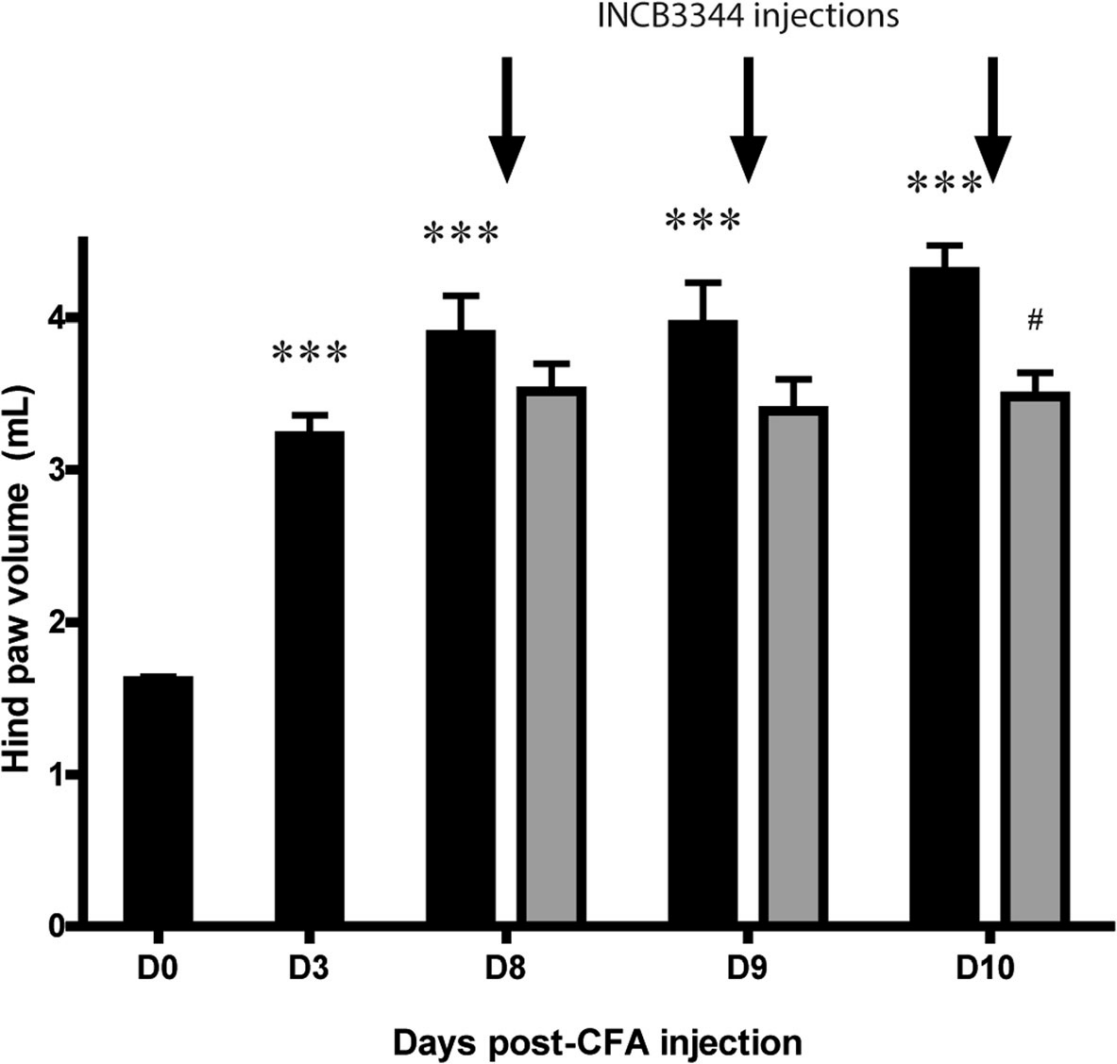
Central blocking of CCR2 activation reduces peripheral inflammation induced by CFA. The ipsilateral hind paw volume was measured by plethysmometry before and 3, 8, 9, and 10 days after CFA injection. Starting on day 8, animals received a daily *i.t.* injection of either vehicle (DMSO 6%) or INCB3344 1 h prior to edema measurement. Black bars represent vehicle-treated CFA animals whereas gray bars correspond to INCB3344-treated CFA animals. * Relates to baseline versus vehicle comparisons, # to vehicle versus INCB3344. Data correspond to mean ± SEM (*n* = 8 for each group). *^, #^*P* < 0.05, ***P < 0.001; two-way ANOVA followed by Bonferroni post-tests

The inflammation primed by CFA also resulted, at the peripheral level, in a progressive swelling only of the ipsilateral hind paw. The hind paw doubled in size on day 3 and was still slightly increasing at day 8 post-CFA (*P* < 0.001, Fig. 3). More importantly, INCB3344-injected rats exhibited a 31% decrease in their paw volume on the third day of treatment, compared to their vehicle-treated counterparts (*P* < 0.01).

### Repeated i.t delivery of a CCR2 antagonist reduces the expression level of pronociceptive markers promoted by CFA

**Fig. 4:**
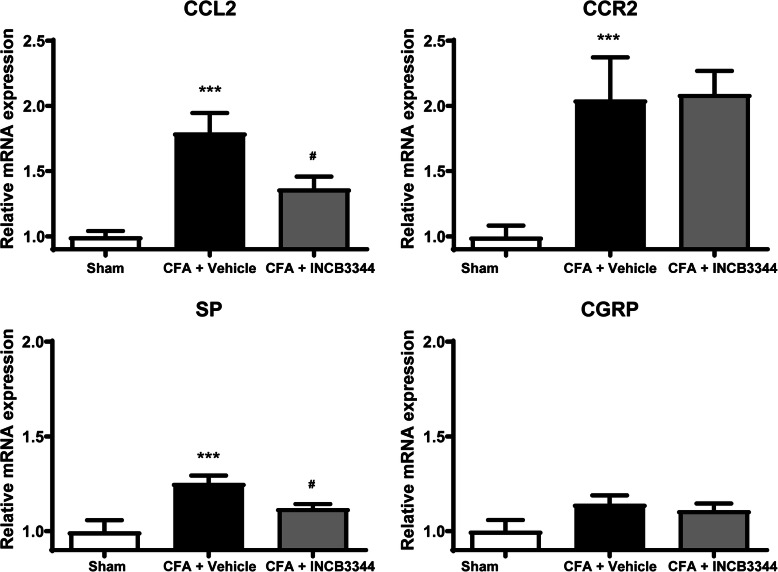
Repeated INCB3344 treatment inhibits the expression of various pro-nociceptive peptides. mRNAs were extracted from ipsilateral rat DRG L4-L6 on day 10 post-CFA, after three daily *i.t*. injection of vehicle (DMSO 6%) or 45 μg/kg INCB3344. Relative expression of each gene of interest was obtained by qPCR using the standard curve method. Results represent the fold increase in mRNA expression induced by CFA with or without treatment with INCB3344. Data are expressed as mean ± SEM (*n* = 8 animals for each group). ***P < 0.001 when comparing the Sham group with the CFA + vehicle group. ^#^*P*<0.05 when comparing CFA + INCB3344 to CFA + vehicle; one-way ANOVA followed by a Bonferroni’s post-test

We next examined whether inhibition of the CCL2-CCR2 axis was able to modify the expression patterns of different pronociceptive markers in rats receiving CFA. To this aim, we measured the changes in CGRP and SP expression as well as CCL2, CCR2 by qPCR using mRNA extracted from ipsilateral DRG L4-L6. Following CFA administration, we observed a significant increase in SP, CCR2, and CCL2 mRNA levels, with no significant change in CGRP expression (*P* < 0.01, Fig. 4). Importantly, repeated *i.t.* treatment with INCB3344 reduced by approximately 50% the CFA-induced increase in CCL2 and SP mRNA (*P* < 0.05, Fig. 4). It had, however, no effect on CCR2 mRNA expression.

### The calcium mobilization elicited by CCL2 under chronic inflammation is blocked by a CCR2 antagonist

**Fig. 5:**
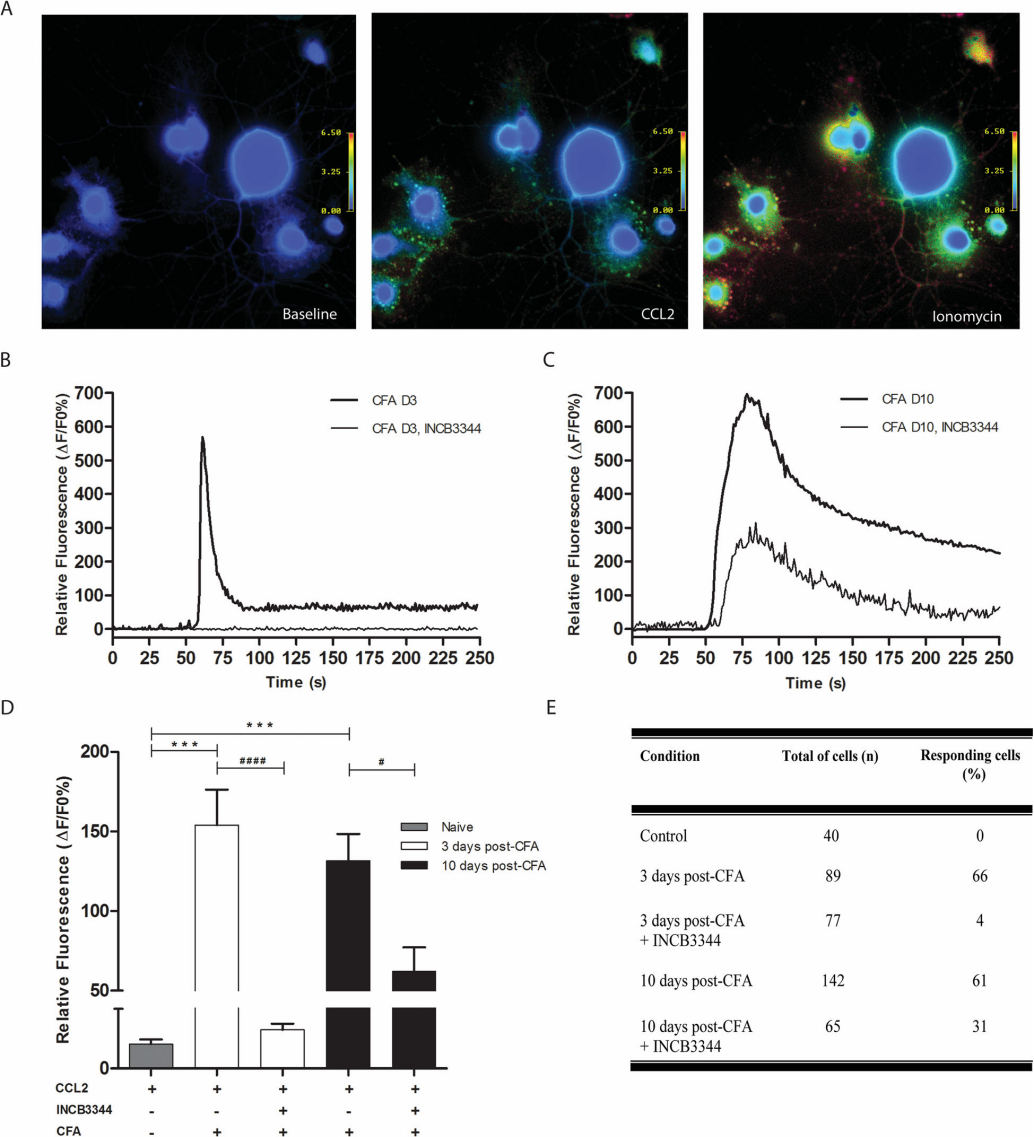
Blocking CCR2 with INCB3344 inhibits calcium-mobilization induced by CCL2 in DRG neurons from CFA rats. DRG neuronal cells were loaded with 2 μm Fura-2-AM and monitored for relative fluorescence changes. **a** Representative images of calcium imaging recording of DRG cell culture from rats, 3 days post-CFA. Digital images (×40) were taken immediately before addition of CCL2 (left), after the addition of 50 nM CCL2 (middle), and after treatment with 1 μM of the calcium ionophore ionomycin, used as a positive control (right). Graded color scales indicate Fura-2-AM emission ratios (340/380 nm), with purple representing low [Ca^2+^]_i_ and yellow/red representing high [Ca^2+^]_i_. Pre-incubation of 100 nM INCB3344 (30 min) blocks the rise of calcium induced by CCL2 application at day 3 post-CFA (**b**) and partially inhibits calcium-released at day 10 post-CFA (**c**). Quantification of the calcium mobilization induced by CCL2 in the presence or absence of INCB3344 (**d**). Percentage of cells responding to CCL2 in the presence or absence of INCB3344 (**e**). Cells were considered responsive when we observed at least 30% of increase in fluorescence intensity ratio, compared to baseline. Data shown correspond to mean ± SEM. ^#^ *P* < 0.05, *** *P* < 0.001 ^####^ *P* < 0.0001; one-way ANOVA followed by Bonferroni post-test

To investigate whether the development of inflammatory pain induced changes in DRG neuron responsiveness to CCL2, we compared the effect of CCL2 stimulation on calcium mobilization in primary cell culture from naïve rats and from rats exposed for 3 or 10 days to CFA. We observed significant elevation in Ca^2+^ mobilization as determined by the change in ratio of fura-2-AM fluorescence, when sensory neurons from CFA animals were treated with 50 nM of CCL2 compared to those of naïve rats (*P* < 0.001) (Fig. 5a–d). Furthermore, considering the cellular response as more than 30% of fluorescence change from baseline, we did not observe [Ca^2+^]_i_ response to CCL2 in DRG neurons from naïve animals, as compared to CFA-treated rats (Fig. 5e).

We next assessed if the blocking of CCR2 by pre-incubating INCB3344 prior to CCL2 stimulation reduced the calcium mobilization. At day 3 post-CFA, only 4% of INCB3344-treated neuronal cells responded to CCL2 compared to 66% in absence of the antagonist (Fig. 5d, e). Furthermore, inhibition of CCR2 on day 10 post-CFA was less effective in reducing the [Ca^2+^]_i_ level. Indeed, 31% of cells were still able to respond to CCL2 in the presence of INCB3344.

### CCL2 release is increased under inflammatory pain condition

**Fig. 6:**
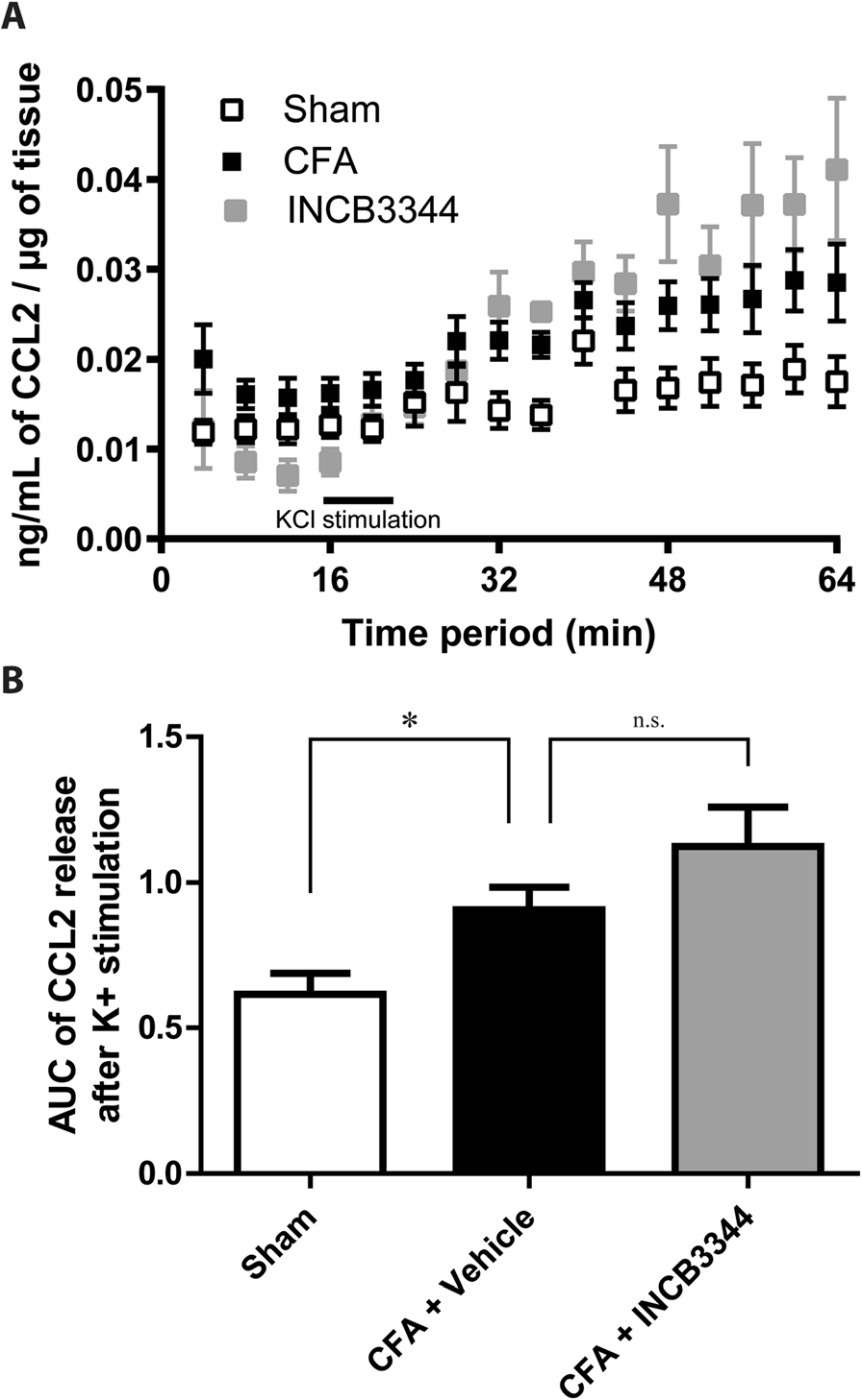
CCL2 release in DRG is not affected by chronic INCB3344 treatment. **a**, **b** Ipsilateral DRGs L4-L6 were extracted on day 10 post-CFA, after three daily *i.t.* injection of vehicle or 45 μg/kg INCB3344. Levels of CCL2 in the superfusate were determined by ELISA. Data in (**b**) correspond to the mean AUC ± SEM (*n* = 8 for each group) of CCL2 release on 11 collected fractions over 44 min after K^+^ stimulation (from 20 to 64 min). *P < 0.05; one-way analysis of variance followed by Bonferroni post-test

To determine the underlying mechanisms by which blockade of CCR2 reduces the course of inflammatory pain, we measured the release of CCL2 by ipsilateral rat L4-L6 DRGs, 10 days following CFA injection. We observed a 1.5-fold increase in CCL2 release following K^+^ depolarization (Fig. 6, *P* < 0.05). However, daily injections of 45 μg/kg INCB3344 for 3 consecutive days failed to reverse the CFA-induced CCL2 release.

### Repeated treatment with INCB3344 blocks the anterograde transport of substance *P* induced by CFA treatment

**Fig. 7:**
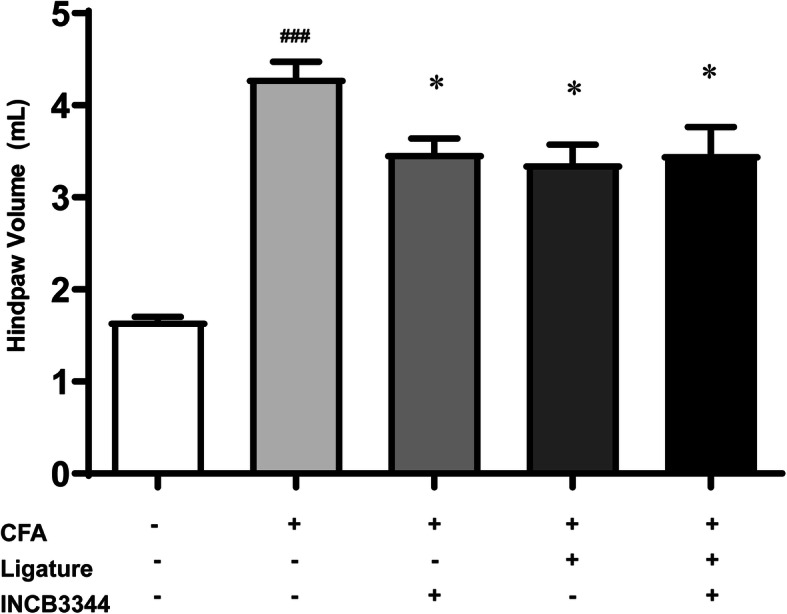
Tight ligature of the ipsilateral sciatic nerve reduces peripheral edema. Eight days after intraplantar administration of CFA, anterograde transport toward the periphery was blocked by a tight constriction of the sciatic nerve. Animals received three daily injection of INCB3344 or vehicle starting on day 8 post-CFA, 12 h before sciatic surgery. Hind paw volume was measured 48 h after the sciatic ligature. Data shown corresponds to mean ± SEM (*n* = 6 for each group). ^###^ *P* < 0.001 when comparing sham animals with CFA untreated and non-ligatured rats. *P < 0.05 when compared to CFA untreated and non-ligatured animals (one-way analysis of variance followed by Bonferroni post-test, only comparing with the CFA alone group)

We further investigate the possible relation between CCL2-induced calcium mobilization and the development of peripheral inflammation associated with CFA. To this aim, we blocked the peripheral anterograde transport through a tight sciatic nerve ligation at day 8 post-CFA. Forty-eight hours after the surgery, we found that the ligature caused a reduction in the ipsilateral hind paw volume in CFA animals compared to non-ligatured CFA-treated rats (Fig. 7, *P* < 0.05). However, spinal INCB3344 delivery did not further reduce the hind paw edema.

**Fig. 8:**
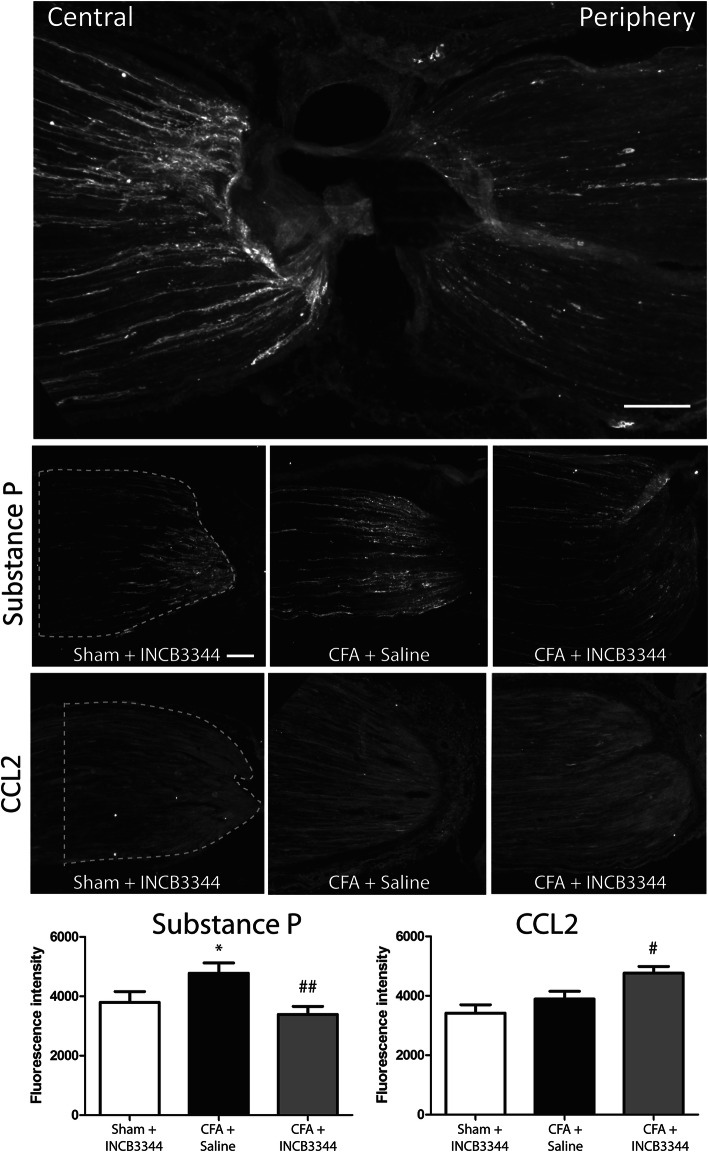
Anterograde transport of SP is reversed by INCB3344 treatment in CFA-treated animals. Ligature of the ipsilateral sciatic nerve was performed 8 days after CFA or saline administration. Forty-eight hours after ligature, the sciatic nerve was harvested for immunohistochemistry. A representative slice of SP immunoreactivity (IR) can be seen in the upper panel, showing both peripheral and central side of the constriction site. Representative photomicrographs of the central accumulation of SP-IR or CCL2-IR in sham or CFA animals, with or without INCB3344 treatment can be seen in the following smaller insets. The fluorescence intensities of the central side of SP and CCL2-IR were also quantified by measuring the fluorescence intensity in the selected region of interest (ROI). Scale bar represents 60 μm. Data correspond to mean ± SEM. We analyzed 10 nerve sections per animals with three animals per condition. *P < 0.05 compared to Sham + INCB3344. ^#^*P* < 0.05, ^##^*P* < 0.01 compared to CFA + saline; one-way analysis of variance followed by Bonferroni post-test

We next determined whether the peripheral transport of CCL2 or of the neurogenic mediators, substance P and CGRP was affected following repeated INCB3344 treatment. We found a 26% higher accumulation of SP labelling at the central side of the ligature in rats receiving CFA, compared to sham animals treated with INCB3344 (Fig. 8, *P* < 0.05). In sharp contrast, we did not detect any change in CCL2 accumulation. Importantly, repeated INCB3344 treatment reverted the accumulation of substance P back to the level of sham rats (*P* < 0.01), while also increasing CCL2 accumulation (*P* < 0.05). No difference in CGRP accumulation could be observed.

## Discussion

Historically, pain among patients developing inflammatory arthritis has been thought to be mainly attributed to peripheral inflammation. This has notably led to the development of a new class of therapeutics acting on key tissues to decrease inflammation, called DMARDs [33]. Although conventional DMARDs indirectly manages pain symptoms by slowing down the disease progression, patients still report feeling moderate to severe pain [34]. Recently, more attention has therefore been directed towards the treatment of pain itself, with the development of new analgesic agents targeting peripheral nociceptive pathways, such as CNTX-4975, a synthetic form of capsaicin which selectively targets the TRPV1 receptor or Tanezumab, an anti-nerve growth factor (NGF) monoclonal antibody. The promising analgesic results obtained in recent phase III clinical trials with these new treatment options further encourage the development of new pharmacological strategies that successfully target nociceptors [57].

**Fig. 9:**
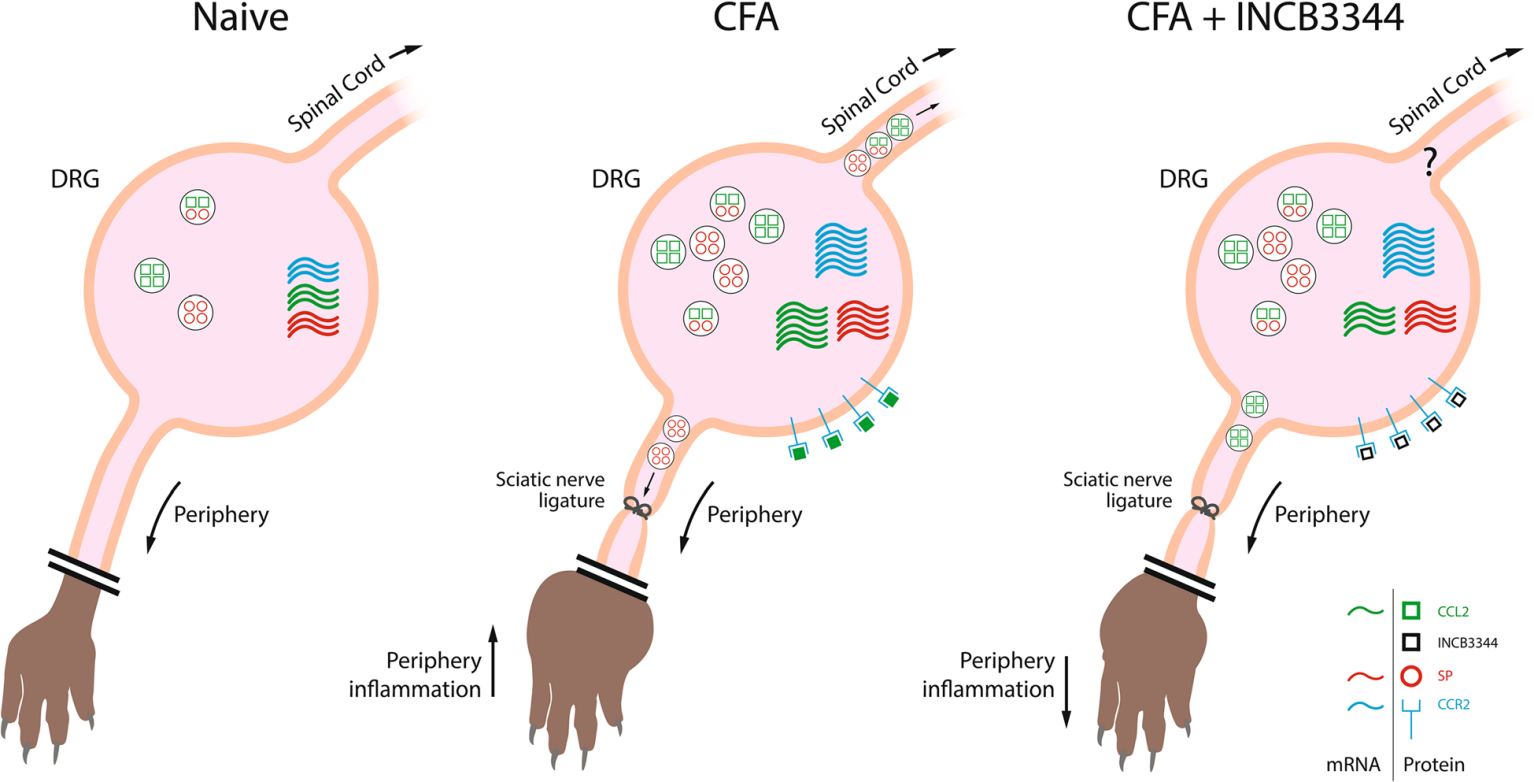
Schematic representation of the regulation of the CCL2-CCR2 axis in dorsal root ganglion (DRG) following peripheral inflammation. CFA-induced inflammation provokes an increase in CCL2, CCR2, and SP mRNA expression in the ipsilateral DRG. The higher expression of CCR2 facilitates intracellular calcium mobilization induced by CCL2 and increases the release of SP and CCL2 toward the spinal cord, as well as the anterograde transport of SP toward the periphery. As shown, CCL2 can be packaged into SP-containing vesicles as well as SP-free vesicles. These two CCL2-containing populations of secretory vesicles can therefore be independently transported peripherally to the injury site and centrally to the dorsal spinal horn. By preventing CCR2 activation through repeated administration of the CCR2 antagonist, INCB3344, we found a marked reduction of the enhanced expression of CCL2 and SP mRNA, a decrease in CCR2-mediated intracellular calcium mobilization and a subsequent reduction of SP peripheral release, which significantly reduce neurogenic inflammation and hind paw edema. Altogether, preventing CCR2 activation at the DRG level contributes to reduce painful hypersensitivity and neurogenic inflammation

Among the therapeutic options, inhibition of the CCL2/CCR2 chemokine axis holds great promise for controlling chronic painful arthritis. Indeed, the CCL2/CCR2 signaling has been found to play key roles in peripheral and spinal nociceptive processing, mediating nociceptor sensitization and increase in the synaptic transmission in the spinal dorsal horn [4, 9, 11, 18, 58]. Standing at the crossroads of the immunobiology and neurobiology, the CCL2/CCR2 chemokine system is also able to trigger peripheral inflammation at the distal site, to promote neuron-glia interaction, and to orchestrate the neuroinflammation response through the recruitment of peripheral T cells and monocytes and/or activation of resident glial cells [12, 16]. Here, we provide significant mechanistic insights into the role of the CCL2/CCR2 signaling within the DRG in the development of peripheral inflammation, nociceptor sensitization and pain hypersensitivity. As schematically represented in Fig. 9, peripheral tissue injury followed by intraplantar injection of CFA, which results in paw edema and inflammation induces an increase in CCL2/CCR2 and SP expression in ipsilateral DRGs. This upregulation is accompanied by an enhanced excitability of primary nociceptive neurons on days 3 and 10 post-CFA, as revealed by the CCR2-dependent increase in intracellular calcium mobilization following CCL2 stimulation. As shown using the ex vivo superfusion of DRG explants of CFA-treated rats, this is followed by a calcium-dependent release of CCL2. Finally, the excitation and sensitization of nociceptors following peripheral inflammation drives the anterograde transport of SP at their peripheral nerve terminals as well as paw swelling. Importantly, our results highlight that blockade of the CCL2/CCR2 signaling following repeated i.t. administration of the CCR2 antagonist, INCB3344 reduces the expression of both CCL2 and SP in DRGs of CFA-treated rats, dampens sensory neuron excitability by limiting the intracellular calcium mobilization, and subsequently decreases peripheral transport and release of SP at the terminal nerve endings. Then, this pharmacological inhibition of CCR2 significantly reduces the neurogenic inflammation as well as the stimuli-evoked and movement-evoked nociceptive behaviors in CFA-treated rats.

The results of the present study reinforce the previous concept on the neuromodulator/neurotransmitter role of CCL2 [58, 59]. Indeed, our data support the idea that CCL2, synthesized and released by the soma of DRG neurons, directly excites sensory nociceptive neurons by autocrine and/or paracrine processes under peripheral chronic inflammation. Accordingly, CCL2-positive DRG neurons were found to be increased in inflamed rat tissues [60, 61]. The specific downstream signaling pathways by which CCL2 drives the neuronal hyperexcitability under chronic inflammatory pain conditions yet remain to be uncovered. However, since we demonstrate that following acute (D3) or chronic (D10) CFA treatment, CCL2 elicits a greater calcium elevation in sensory neurons through a CCR2-dependent mechanism compared to naïve DRG neurons, we can hypothesize that functional changes in extracellular calcium influx and/or CCL2-induced calcium release from internal stores might underlie CCL2-induced neuronal activation. In that sense, it has been shown that inhibition of N-type Ca^2+^ channels by the omega-conotoxin GVIA channel blocker or treatment with either thapsigargin or ryanodine significantly reduced CCL2-induced intracellular calcium influx and the concomitant release of CGRP by primary sensory neurons [62]. Other modes of action of CCL2/CCR2 could also drive the neuronal hyperexcitability. Indeed, we and others have demonstrated that CCL2 enhanced sensory neuron excitability by increasing the functional activity of tetrodotoxin-resistant (TTX-R) sodium channel Na_v_1.8 currents [56, 63]. This effect is CCR2-mediated as treatment with INCB3344 blocked the potentiation of Na_v_1.8 currents by CCL2 in a concentration-dependent manner [56, 64]. Similarly, the increase in CCR2/CCL2 signaling observed following tissue inflammation could cause the peripheral sensitization of DRG nociceptive neurons and drive the hyperalgesic state by upregulating the expression and function of the capsaicin-sensitive TRPV1 ion channel [22, 59, 63]. This idea is supported by the presence of CCL2/CCR2 within TRPV1-expressing sensory neurons [14, 59, 60]. In turn, as demonstrated in in vitro superfusion assay, capsaicin evokes calcium-dependent release of CCL2 [14, 22] and TRPV1 inhibition decreases CCL2-induced hyperalgesia [65]. Collectively, these results demonstrate that the CCL2/CCR2 axis and TRPV1 may act in unison to sensitize nociceptors.

To gain further insights into the mechanisms by which CCR2 activation induces inflammatory hypernociception, we determined whether the increase in intracellular calcium mobilization was translated into a greater CCL2 secretion by DRG explants from CFA-treated rats. As previously observed in naïve and neuropathic animals [14, 22], K^+^ stimulation inducing neuronal depolarization increases CCL2 release from CFA-exposed DRGs compared to controls. It is widely accepted that extracellular calcium influx and calcium-induced calcium release (CICR) from internal stores play an important role in the release of the pro-nociceptive neuropeptides SP and CGRP from nociceptors [62, 66]. Therefore, since CCL2 stimulates intracellular calcium elevation through both ryanodine-sensitive calcium stores and N-type Ca^2+^ channels, the release of CCL2 from CFA-exposed DRGs may thus influence the transport and release of these pain-related neuropeptides by presynaptic mechanism. Accordingly, our results demonstrate for the first time that the anterograde transport of SP (but not CGRP) towards the peripheral nerve terminals was inhibited by blocking CCR2 using INCB3344. Interestingly, despite the increased expression and release of CCL2 by CFA-exposed nociceptors, immunohistochemical analysis of ligated nerves does not reveal any increase in CCL2 immunoreactivity in CFA-treated animals, ruling out the contribution of a DRG-derived CCL2 release toward the periphery, at least at day 10 post-CFA. This is consistent with the demonstration that CCL2 is locally produced at the inflammation site by macrophages/monocytes in CFA inflamed rats and that treatment with INCB3344 dose-dependently inhibits macrophage influx [49, 67]. As previously shown [22], CCL2 is probably conveyed to the terminals of nociceptors and released at the spinal dorsal horn to modulate the activity of post-synaptic neurons and surrounding glial cells. Although we did not further investigate the cellular mechanisms behind the relation between CCL2 and SP, CCR2 is known to sensitize TRPV1 [59], whose stimulation induces the release of SP from sensory nerve fibers [68, 69]. Surprisingly, *i.t*. treatment with INCB3344 does not induce a decrease in the anterograde transport of CCL2 and even increases its accumulation centrally to the sciatic ligature. This seems to indicate that chronic blockade of CCR2 activation leads to increased CCL2 expression, as previously reported in a clinical study in patients with advanced solid tumors treated with an anti-CCL2 human monoclonal antibody [70].

Primary afferent neurons can directly contribute to peripheral inflammation and immune cell recruitment through the release of neuropeptides, such as SP and CGRP [71, 72]. As superfusion experiments does not distinguish between CCL2 release toward the spinal cord or the periphery, we initially thought that CCL2 would be concomitantly released with SP and/or CGRP following CFA intraplantar administration, thus contributing to the neurogenic inflammation process. Contrary to SP, CCL2 does not seem to be transported to the peripheral inflammation site. Accordingly, we and others previously reported that CCL2 can be packaged into SP-containing vesicles as well as SP-free vesicles [14, 59]. This might suppose that these two CCL2-containing populations of secretory vesicles can be alternatively released in response to nociceptive signals and then enhanced nociceptor sensitization and pain hypersensitivity. Interestingly, our results also reveal that the nerve ligation reduces peripheral inflammation, indicative of a contribution of neurogenic inflammation to the CFA-induced peripheral edema. Moreover, the reduction in hind paw volume was similar to INCB3344-treated rats, suggesting that CCR2 activation at the DRG level contributes to peripheral inflammation, probably through the release of SP. Accordingly, administration of NK1 antagonists (i.e., the main SP receptor) reduces the plasma extravasation induced by intra-articular administration of carrageenan [73, 74]. Finally, as joint inflammation directly contributes to pain [28], this peripheral reduction in swelling could contribute to the observed analgesic efficacy of INCB3344.

Here, we further unveil the therapeutic potential of a CCR2 antagonist to relieve the pain behaviors associated to painful inflammatory conditions. We first demonstrated that CCL2 exerted a pronociceptive action in the formalin inflammatory phase through the exacerbation of the pain-related behaviors induced by a mild injection of formaldehyde. As expected, co-administration of the CCR2 antagonist completely prevented CCL2-induced pain hypersensitivity, thus indicating that CCL2 elicited pain facilitation via a CCR2-dependent mechanism. Similarly, mice overexpressing CCL2 were hypersensitive to chemical-induced nociception [75], while mice deficient for CCR2 displayed decreased nociception in the inflammatory phase of the formalin test [19, 75]. Accordingly, intracisternal administration of CCL2 also facilitated formalin-induced scratching behavioral responses in the orofacial area [76]. Mechanistically, it is well accepted that the inflammatory phase of the formalin test results from the combination of activation of primary afferent fibers by peripheral inflammatory mediators and functional changes in the dorsal spinal horn, notably through NMDA and NK1 receptor activation, thus leading to central sensitization [77]. Interestingly, in addition to sensory neuron modulation, CCL2 participates in central sensitization by potentiating the activity of NMDA receptor currents in CCR2-expressing excitatory neurons located in lamina IIo of the spinal dorsal horn under peripheral inflammation [78–80]. Thus, these results further emphasize that CCL2 is anterogradely transported by primary afferent neurons to be released in the spinal dorsal horn in inflamed rats.

Chronic pain in patients with RA or OA leads to important physical distress as well as to the loss of patients’ autonomy and quality of life. Weight bearing activities are the main source of severe pain episodes in people suffering from arthritis [81, 82]. In addition, it is clinically demonstrated that most pharmacological agents relieve pain at rest while being less effective on movement-evoked pain [83]. Here, we demonstrated that repeated i.t. injection of INCB3344 exerted anti-allodynic effects, as previously observed in neuropathic, postoperative, and cancer-induced bone pain models using anti-CCL2 antibodies or small molecule antagonists of CCR2 [15, 84–91]. In contrast, inhibition of CCR2 using intraplantar or subcutaneous injection of the antagonist RS504393 was only effective in reversing thermal hyperalgesia in CFA inflamed mice [67]. Importantly, this decrease in mechanical hypersensitivity was accompanied by a reduction in the ambulation-evoked pain behaviors in freely moving CFA-treated rats. Indeed, in line with clinical reports, CFA-induced paw inflammation generated an important load redistribution on the contralateral non-injured limb. Following repeated administration with INCB3344, we observed a partial recovery of the weight borne on the ipsilateral limb. This reversal in pain-induced weight redistribution was of a similar extent than the reversal in stimulus-evoked mechanical allodynia. Interestingly, Longobardi et al. recently showed that systemic blockade of CCR2 by RS504393 improved the weight redistribution in a murine model of injured-induced OA (i.e., destabilization of medial meniscus; DMM) [92]. Likewise, mice invalidated either for CCL2 or CCR2 exhibited less pain-related behaviors post-DMM [93].

## Conclusion

Altogether, these results identify a promising dual action of CCR2 blockade, acting on both nociceptor sensitization and peripheral inflammation, that could lead to the development of more adequate pharmacological agents to manage chronic painful arthritis.

## Data Availability

The datasets used and/or analyzed during the current study are available from the corresponding author on reasonable request.

## Abbreviations

CCL2: Chemokine (C–C motif) ligand 2
CCR2: CC chemokine receptor 2
MCP-1: Monocyte chemoattractant protein 1
CFA: Complete Freund’s adjuvant
RA: Rheumatoid arthritis
OA: Ostearthritis
DRG: Dorsal root ganglia
SP: Substance P
CGRP: Calcitonin gene-related peptide
i.t.: Intrathecal
DMARD: Disease-modifying antirheumatic drugs
TRPV1: Transient receptor potential cation channel subfamily V member 1
NK1: Neurokinin 1
NMDA: *N*-methyl-d-aspartate
DMM: Destabilization of medial meniscus
